# Traditional Chinese Medicine for Hashimoto’s Thyroiditis: Focus on Selenium and Antioxidant Phytochemicals

**DOI:** 10.3390/antiox13070868

**Published:** 2024-07-19

**Authors:** Sheng Huang, Panos G. Ziros, Dionysios V. Chartoumpekis, Georgios Psarias, Leonidas Duntas, Xinhe Zuo, Xinyi Li, Zhiguo Ding, Gerasimos P. Sykiotis

**Affiliations:** 1 Department of Thyropathy, Dongzhimen Hospital, Beijing University of Chinese Medicine, Beijing 100700, China; sheng.huang@chuv.ch; 2 Service of Endocrinology, Diabetology and Metabolism, Lausanne University Hospital and University of Lausanne, 1011 Lausanne, Switzerland; panos.ziros@chuv.ch (P.G.Z.); dionysios.chartoumpekis@chuv.ch (D.V.C.); georgios.psarias@chuv.ch (G.P.); 3 Unit of Endocrinology, Metabolism and Diabetes, Evgenideion Hospital, National and Kapodistrian University of Athens, 11528 Athens, Greece; leonidas@duntas.gr; 4 Thyroid Disease Diagnosis and Treatment Center, Hubei Provincial Hospital of Traditional Chinese Medicine, Wuhan 430074, China; zxh31047@stmail.hbucm.edu.cn; 5 Department of Traditional Chinese Medicine and Rehabilitation, Beijing Health Vocational College, Beijing 101101, China; 20170935056@bucm.edu.cn; 6 Department of Thyropathy, Sunsimiao Hospital, Beijing University of Chinese Medicine, Tongchuan 727100, China

**Keywords:** Hashimoto’s thyroiditis, traditional Chinese medicine, clinical implication, related active compound, antioxidant, anti-inflammation, molecular mechanism

## Abstract

Hashimoto’s thyroiditis (HT) is not only the most frequent autoimmune thyroid disease (AITD), but it also has a significant impact on patients’ health-related quality of life (HRQoL), and it has been variably associated with differentiated thyroid carcinoma. Even though its pathogenesis is still incompletely understood, oxidative stress is believed to play an important role. Hypothyroidism related to later stages of HT can be treated with levothyroxine substitution therapy; various approaches such as selenium supplementation and iodine-restricted diets have been proposed as disease-modifying treatments for earlier stages, and even thyroidectomy has been suggested for refractory cases of painful HT. Nevertheless, many patients still report suboptimal HRQoL, highlighting an unmet medical need in this area. The concepts and approaches of traditional Chinese medicine (TCM) in treating HT are not broadly known in the West. Here, we provide an overview of TCM for HT, including combinations of TCM with selenium. We encompass evidence from clinical trials and other studies related to complex TCM prescriptions, single herbs used in TCM, and phytochemicals; wherever possible, we delineate the probable underlying molecular mechanisms. The findings show that the main active components of TCM for HT have commonly known or presumed antioxidant and anti-inflammatory activities, which may account for their potential utility in HT. Further exploring the practices of TCM for HT and combining them with evidence- and mechanism-based approaches according to Western standards may help to identify new strategies to alter the clinical course of the disease and/or to treat patients’ symptoms better and improve their HRQoL.

## 1. Introduction

Hashimoto’s thyroiditis (HT), also called chronic lymphocytic thyroiditis, is the most frequent cause of subclinical or overt hypothyroidism in areas with sufficient iodine intake [1]. Its histological hallmarks include diffuse lymphocytic infiltration, especially by T cells, and destruction of the follicular structures of the thyroid parenchyma [2]. Serologically, HT is characterized by elevated levels of serum autoantibodies against proteins central to thyroid follicular cell function, such as anti-thyroid peroxidase (anti-TPO) autoantibodies (TPOAb) and anti-thyroglobulin (anti-TG) autoantibodies (TGAb) [3,4]. The natural course of HT can be typically divided into three stages [5]. Patients may initially experience a self-limited transient thyrotoxicosis, which is due to the release of stored thyroid hormones from damaged thyroid follicles. The second stage is distinguished by a euthyroid phase, in which the thyroid tissue that has been retained compensates for the decreased secretory capacity resulting from the damage to follicles. When the progressively affected thyroid gland does not secrete sufficient thyroid hormone to compensate, it enters a third stage, resulting in hypothyroidism. Thus, although patients can be euthyroid or even present with thyrotoxicosis at initial diagnosis, it is believed that most HT cases ultimately evolve naturally towards hypothyroidism [6].

### 1.1. Epidemiology and Symptomatology

HT is the most prevalent autoimmune thyroid disease (AITD). The overall prevalence of HT in adults is 7.5%; similar to other autoimmune diseases, the prevalence of HT is higher in women than in men (17.5% vs. 6.0%, respectively) [7]. Seropositivity for thyroid autoantibodies has been associated with adverse pregnancy outcomes, even when thyroid function is normal [8,9,10]. Moreover, various studies have suggested HT as a potential risk factor for differentiated thyroid carcinoma [11]. Although still largely unclear, the cause of HT is related to a combination of genetic susceptibility and environmental factors that together provoke the loss of immunological tolerance, with a consequent autoimmune attack on thyroid tissue [3]. Clinically, the diagnosis of HT is usually made by documenting the presence of thyroid autoantibodies in the serum and/or the presence of a typical heterogeneous pattern on thyroid ultrasound [6]. Patients with HT may report various local or systemic clinical symptoms, such as profound fatigue, poor sleep quality, neck swelling, dysphagia, mood disorders, etc. [12]. Although it is not yet firmly established whether the links between such symptoms and the presence of thyroid autoimmunity are causal and/or independent of thyroid function, a diagnosis of HT can be correlated with a significantly decreased health-related quality of life (HRQoL) [13,14,15]. Taken together, the above considerations regarding the prevalence and clinical correlates of HT highlight this disease as an area of unmet medical need with broad relevance for public health.

### 1.2. Brief Description of Underlying Molecular Mechanism in HT

**Figure 1:**
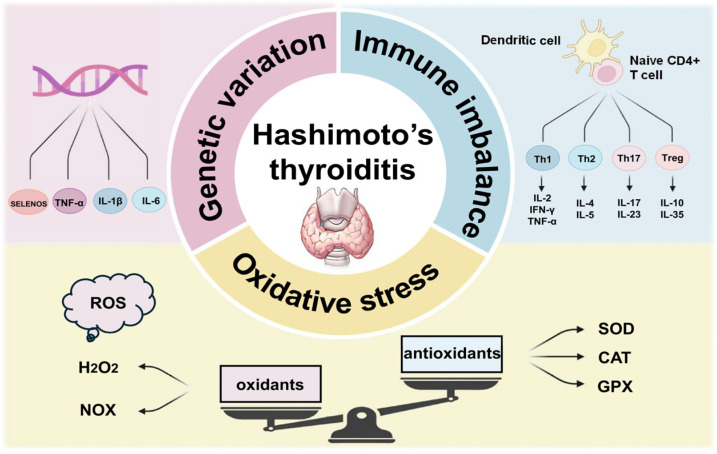
Graphical summary of some of the main underlying molecular mechanisms in HT. Genetic variation refers to single nucleotide polymorphisms (in genes such as *SELENOS*, *TNF-α*, *IL-1β*, *IL-6*) that increase the risk of HT. Immune imbalance refers to the production of cytokines by Th lymphocytes that induce thyroid follicular cells to express surface Human Leukocyte Antigen DR (HLA-DR), thereby making them susceptible to immune attack. Part of the immune imbalance is related to the deficient ability of Tregs to inhibit the activity of immune cells targeting the thyroid. Oxidative stress refers to the excess production of ROS and RNS that can not be counterbalanced by the thyrocytes’ antioxidant systems.

The pathogenesis of HT is not completely understood; nevertheless, three factors that contribute to its development are oxidative stress, immune imbalance, and genetic variation, promoting a cascade of thyroid follicle destruction [16,17,18] (Figure 1).

#### 1.2.1. Oxidative Stress

Oxidative stress is a cellular state that ensues when there is excessive accumulation of pro-oxidant substances, such as reactive oxygen species (ROS), reactive nitrogen species (RNS), and other free radical species; it is a factor known to contribute to the pathogenesis and progression of diverse autoimmune diseases, including HT [19,20,21]. Oxidative stress leads to structural damage to biomolecules, and it undermines the genomic stability of the cell. Among other effects, oxidative stress accelerates the formation of advanced glycation end products (AGEs), a complex mixture of modified and crosslinked proteins [22]. The body is equipped with an antioxidant defense system that guards against oxidative damage caused by these reactive oxidants and plays a major role in protecting cells from oxidative stress and damage. Cells dispose of various endogenous antioxidant enzymes to counter oxidative stress and to protect lipids, proteins, and DNA from damage; these include superoxide dismutase (SOD), glutathione peroxidase (GPx), catalase (CAT), and many others [23]. A study showed that in euthyroid HT patients, the oxidative/antioxidative balance in the serum is shifted toward the oxidative side; the possible significant involvement of AGEs in HT is also reported [19].

Hydrogen peroxide (H_2_O_2_) is a relatively stable ROS that is a key element in thyroid hormone biosynthesis [24,25]. The participation of NADPH oxidases (NOX) in the production of H_2_O_2_ for thyroid hormone synthesis may be associated with the pathophysiology of AITD, potentially through interactions of oxidants with TPO and TG that alter their activity and promote their immunogenicity [26]. Excessive intake of iodine is considered a risk factor for the development of AITD, possibly because it enhances ROS production while also reducing the levels of endogenous antioxidants [27,28,29]. In HT patients, TPOAb correlates negatively with the serum levels of glutathione, the main cellular antioxidant, and TPOAb and TGAb antibodies correlate positively with serum biomarkers of oxidative status, such as total oxidant status (TOS) and oxidative stress index (OSI) [24,30].

#### 1.2.2. Immune Imbalance

In response to exposure to various stimuli, naive CD4+ T cells differentiate into various subsets of T helper (Th) cells, including Th1, Th2, Th17, and T regulatory lymphocytes (Tregs). They play a key role in the pathogenesis of inflammatory and autoimmune diseases by producing distinctive sets of cytokines [31]. In HT, Th lymphocytes produce cytokines that induce thyroid follicular cells to express surface Human Leukocyte Antigen DR (HLA-DR), thereby making them susceptible to immune attack. Different Th cell subtypes secrete various inflammatory cytokines: Th1 cells secrete interleukin-2 (IL-2), interferon-γ (IFN-γ), and tumor necrosis factor-alpha (TNF-α), which act by stimulating cytotoxic lymphocytes and macrophages that cause damage to follicles, leading to their destruction. Moreover, Th1 cells induce plasma cells to produce antibodies against TPO, TG, and other thyroidal proteins; Th2 cells secrete IL-4 and IL-5; and Th17 cells secrete IL-17 and IL-23 [32,33]. In HT, part of the immune imbalance is related to the deficient ability of Tregs to inhibit the activity of immune cells targeting the thyroid. IL-10 secreted by Treg regulates the action of Th17 cells and plays a protective role against damage to the thyroid [34]. Th17/Treg imbalance in AITD has been documented clinically in patients, and the pathogenic roles of Th17 cells and their relevant inflammatory mediators have been addressed in in vitro and in vivo studies [31,35,36].

#### 1.2.3. Genetic Variation

HT is associated with multiple genetic predispositions; consanguinity has been associated with increased susceptibility to different inherited conditions [37]. Several single nucleotide polymorphisms (SNPs) are known to increase the risk of developing HT; much of this genetic variation concerns genes that are either thyroid-specific genes or are involved in autoimmunity, inflammation and/or cellular defense against stress (e.g., IL-1β, IL-6, and TNF) [38]. Thus, HT susceptibility genes contribute directly to the key mechanism underlying the development of organ-specific autoimmunity via the breakdown in self-tolerance. Research has shown that the combined effects of SNPs in the upstream regions of the genes encoding TNF-α, IL-6, and IL-10 may be more decisive to induce functional differences and modify the risk for HT [39]. Another polymorphism that contributes to HT susceptibility resides in the promoter region of the gene encoding selenoprotein S (SELENOS), which is involved in the endoplasmic reticulum stress response and is expressed in multiple tissues, including thyroid follicular cells [40]. Further, this functional SNP interacts with functional SNPs in the promoter of the gene encoding the antioxidant transcription factor NRF2 (nuclear factor erythroid 2-related factor 2) to determine the susceptibility to HT, highlighting a functional cross-talk between these two cellular stress defense pathways [18].

### 1.3. Treatment

Despite HT being an autoimmune disorder, the prevailing treatment approach involves the oral administration of synthetic levothyroxine (L-T4). This therapeutic strategy does not target the underlying autoimmune processes, inflammation, or oxidative stress, nor does it modify the natural progression of the disease. Instead, it aims solely to manage hypothyroidism in the advanced stages of HT. L-T4 replacement therapy is typically a lifelong requirement, with the primary therapeutic goal being the normalization of serum thyroid-stimulating hormone (TSH) levels. This approach is predicated on the assumption that the clinical symptoms of HT are predominantly attributable to hypothyroidism. However, it is noteworthy that patients with HT-induced hypothyroidism may continue to experience persistent symptoms despite achieving normal serum levels of TSH and thyroxine (T4) [14]. Whether infra-physiological serum levels of T3 (triiodothyronine) in patients treated with L-T4 contribute to their symptoms is an interesting topic under investigation that is beyond the scope of the present study [41]. Another hypothesis is that residual or hormone-independent symptoms might be due to the autoimmune, inflammatory, and/or pro-oxidant processes in HT; by extension, in that scenario, disease-modifying approaches could potentially help ameliorate such symptoms. Among these, selenium supplementation warrants special consideration, and it is actively being studied as a potential disease-modifying treatment for HT in both the West and China [42].

#### 1.3.1. Selenium and HT

Selenium is an essential trace element that has a higher concentration in the thyroid than in most other organs [43]. It is an antioxidant substance that contributes to the growth and development of cells and strengthens the immune system. Selenium is found in the structure of selenoproteins (encoded by 25 genes in humans and 24 in mice), which help prevent cellular damage caused by free radicals [44]. It is incorporated into selenoproteins as selenocysteine (Sec), the 21st proteinogenic amino acid, which is codified by a stop codon. In a quite unique manner, specific biosynthetic factors recode the UGA stop codon as Sec [45]. Most of the known selenoproteins are expressed in the thyroid gland, including some with still unknown functions. Among the well-characterized selenoproteins are the iodothyronine deiodinases, glutathione peroxidases, and thioredoxin reductases, enzymes involved in thyroid hormone metabolism, regulation of redox state, and protection from oxidative damage [46,47]. Moreover, SELENOS activity suppresses the transcription of several genes encoding pro-inflammatory cytokines that are involved in the pathogenesis of HT [40,48].

Selenium deficiency has been implicated as a potential factor in the pathogenesis of HT. For example, a study from Iran reported that coexisting selenium deficiency and elevated iodine intake in HT may enhance autoimmune reactions and accelerate the deterioration of thyroid function through oxidative stress [28]. In another study, performed in two counties of Shaanxi Province, China, with close genetic, environmental, and lifestyle similarities but different selenium intake, low selenium status was associated with an increased risk of thyroid disease [49]. A prospective multicenter study in China reported that selenium supplementation significantly reduced TPOAb titers in patients with HT, and it suggested that there may be an important genetic component influencing interindividual differences in the decrease in TPOAb titers [50]. Another Chinese study demonstrated that L-T4 and selenium combination showed more pronounced therapeutic effects compared to L-T4 monotherapy in preventing HT progression, providing a basis for improved prevention and treatment of HT by regulating the Thl/Th2 immune imbalance via selenium supplementation [51].

#### 1.3.2. Traditional Chinese Medicine and HT

In contrast to selenium, which is of interest in both the West and China for the management of HT, the concepts and approaches that are applied in traditional Chinese medicine (TCM) to treat HT are not broadly known in the West. In general, TCM follows the principles of dialectical treatment and holistic concepts. The natural origin of the therapeutic prescriptions, medicinal herbs, and phytochemicals used in TCM are attractive potential advantages in efforts to alter the natural course of non-life-threatening diseases like HT. Even though the literature suggests that TCM can have beneficial effects in patients with HT [52,53], there is still a wide gap between TCM and current Western practices for HT, which is not unlike the situation in other disease areas. In the present review, we aimed to outline current TCM approaches to HT in order to provide a respective reference base, primarily for Western audiences. We outlined the main therapeutic prescriptions, medicinal herbs, and phytochemicals used in TCM for HT, and, wherever relevant, we summarized the associated evidence base for each of them. Finally, to the extent possible, we highlighted the potential underlying mechanisms of benefit in HT, with a particular focus on antioxidant and anti-inflammatory activities.

## 2. Methods

A literature search was performed using the following electronic databases: PubMed, Web of Science, Chinese National Knowledge Infrastructure, Wanfang, and VIP Database. The following search terms (or Chinese database equivalent) were used: (“Hashimoto’s thyroiditis” or “autoimmune thyroiditis” or “autoimmune thyroid disease” or “thyroid peroxidase antibody” or “thyroglobulin antibody” or “thyroid autoantibodies”) AND (“traditional Chinese medicine” or “Chinese herbal medicine” or “Chinese medicine” or ”herb”). Articles were searched from 2000 to 21 February 2024.

The search results were then screened to identify original articles about TCM related to the treatment of HT, mainly including clinical and translational studies related to oral TCM prescriptions, single TCM herbs, and phytochemicals. Studies were excluded if (i) the specified search terms were not present either in the title or the abstract; (ii) the description of the prescription composition was incomplete or uncertain; (iii) they were not included in the Science Citation Index (SCI) and Chinese core journals; (iv) they were published in languages other than English or Chinese; or (iv) the full text was unavailable. TCM names were checked with the Pharmacopoeia of the People’s Republic of China (2020 Edition) [54].

**Figure 2:**
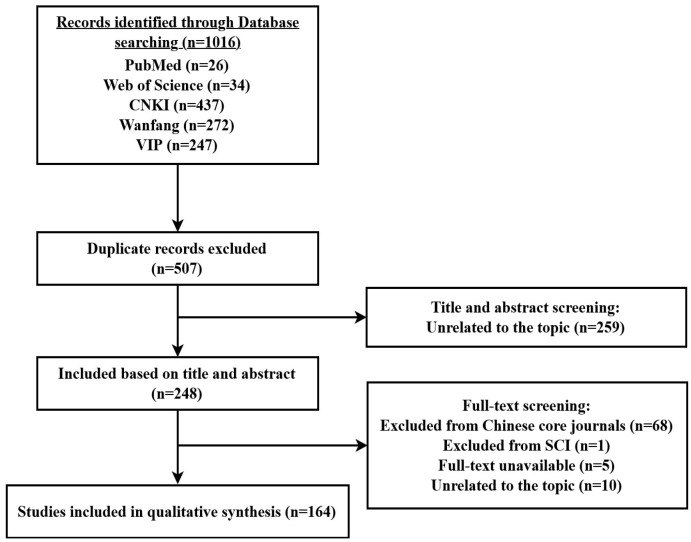
Flowchart of the literature screening.

Following the process indicated in Figure 2, a total of 164 articles were retained; these were studied thoroughly. The most relevant information was used for a qualitative synthesis, and the respective selected articles (a subset of the 164 initially retained) are cited in the following text and tables.

## 3. Clinical and Translational Evidence on TCM for HT

Selenium supplementation can reduce levels of autoimmune thyroid antibodies, which may be beneficial in HT. Nevertheless, the therapeutic effect of selenium on HT has been controversial, and its long-term patient benefits have been questioned. Specifically, four randomized controlled trials (RCTs) that assessed the effects of selenium in adults with HT provided incomplete evidence to support or refute the efficacy of selenium due to unclear to high risk of bias despite the statistically significant decrease in thyroid autoantibodies in some cases [55,56]. Recent data from a follow-up prospective study in Shaanxi Province, China, showed that when iodine was excluded as a confounding factor, low selenium was confirmed as a risk factor for HT, as reflected in a higher TPOAb seroconversion rate in the low-selenium county [57]. Further, a recent systematic review and meta-analysis of RCTs of selenium in HT showed beneficial effects of selenium on various clinical and biochemical parameters associated with HT [58]. This systematic review and meta-analysis did not include a very recent RCT in Denmark that also found a decrease in TPOAb titer following selenium supplementation but no difference in HRQoL between the placebo- and selenium-treated groups [59]. The implications of these latest studies for future research into selenium supplementation for HT have been recently discussed [42].

In China, TCM is extensively utilized as either stand-alone or adjuvant therapy for HT; its use is supported by Chinese studies that exhibit promising efficacy [52,53,60]. A meta-analysis including 18 RCTs involving a total of 1247 patients with HT concluded that Chinese herbal medicine was more efficacious in alleviating clinical symptoms compared to placebo [53]. Similarly, another meta-analysis with 11 RCTs involving 932 patients with HT reported that TCM significantly reduced thyroid antibody titers compared to placebo [61]. In addition, combined therapy of TCM and selenium supplementation exerted a greater beneficial effect on the titer of thyroid autoantibodies and clinical symptoms than selenium supplementation alone [62].

### 3.1. Assessment of Clinical Outcomes in TCM for HT

Clinical outcome measures commonly employed in TCM include the TCM syndrome score and the total effective rate. Given that these concepts may be unfamiliar to Western audiences, we provide a brief explanation to facilitate understanding of the relevant results. Given that the TCM syndrome score and the total effective rate are not standardized across TCM practitioners and clinical studies, typical examples are provided in Table 1 and Table 2, respectively: A typical TCM syndrome score for HT can include anterior neck enlargement, cold intolerance, epigastric pain, emotional instability, fatigue, loss of appetite, sleepiness, insomnia, constipation, and edema. These symptoms are classified as absent, mild, moderate, and severe; they are scored as 0, 2, 4, and 6 points, respectively. In a clinical or research setting, scores are calculated before and during/after treatment, and efficacy is determined according to the % reduction in the score.

In a clinical study, the total effective rate is calculated after classifying participants into the following categories. Clinical cure: the disappearance or near disappearance of clinical symptoms and signs in TCM and reduction in TCM syndrome score by ≥95%. Obvious effect: significant improvement of clinical symptoms and signs, reduction in TCM syndrome score by 70–95%. Effective: improvement of clinical symptoms and signs, reduction in TCM syndrome score by 30–70%. Ineffective: no obvious improvement in clinical symptoms and signs, or even aggravation, and reduction in TCM syndrome score < 30%. The overall effective rate is then calculated according to the following formula: [(number of clinically cured patients + number of patients with obvious effect + number of patients where treatment is effective)/(total number of cases)] × 100%.

### 3.2. Human Studies on Chinese Medicine Prescriptions for HT

It is common that Chinese medicine prescriptions use mixtures of various medicinal herbs with pharmacologically active compounds aiming to achieve additive and/or synergistic effects or to reduce the potentially harmful effects of some pharmacologically active compounds. Several TCM formulas comprising diverse medicinal herbs have been developed for the treatment of HT; the main ones are listed in Table 3 [52,62,63,64,65,66,67,68,69,70,71,72,73,74]. For each one, Table 3 lists respective studies that have shown promising efficacy in HT; according to available reports, adverse effects were rarely observed in clinical practice [63,64,65,72].

In an RCT including 72 patients with HT, treatment with Fuzheng-Jiedu-Xiaoying decoction (200 mL twice daily for 12 weeks) was compared to selenium supplementation (200 μg twice daily for 12 weeks) and was found to significantly improve the clinical symptoms, reduce serum levels of thyroid antibodies, increase the ratio of Treg cells, reduce the ratio of Th17 cells, and reduce the incidence of hypothyroidism, with no observed allergic reactions or other adverse effects on the liver, kidney, or gut [63]. In another RCT enrolling 80 HT patients, Jianpi-Xiaoying decoction (200 mL twice daily for 12 weeks) was more effective compared to selenium supplementation (100 μg twice daily for 12 weeks) at reducing TPOAb and TGAb titers, serum levels of immune factors (IFN-γ, IL-4, IL-4/IFN-γ), and thyroid elasticity. This treatment may thus contribute to the adjustment of the Th1/Th2 immunity imbalance in HT and the alleviation of inflammatory reactions [64]. In an RCT with 60 patients with HT, Qijian-Goiter-eliminating decoction (1 decoction per day divided into two equal doses, twice daily) significantly decreased the levels of TPOAb, TGAb, IL-1β, IL-18 and MDA, and increased the levels of serum SOD, GPx, and total antioxidant capacity (TAC) compared to selenium supplementation (150 μg twice daily for 12 weeks); this treatment then seems to relieve oxidative stress and decrease the level of inflammatory factors [65]. Similarly, Qiaojiafang Granule and Shugan Sanjie formulas were reported to provide more protection against HT compared to selenium supplementation or placebo alone [66,67].

In a study involving 94 patients with HT, treatment with Jieyu-Xiaoying decoction (150 mL twice daily) plus selenium supplementation (200 μg twice daily) for 12 weeks resulted in significant relief of clinical symptoms and a reduction in serum TSH, TPOAb, and TGAb levels, compared to selenium supplementation alone [62]. In another study with 64 HT patients, treatment with Jinkui-Shenqi in the form of a pill (4 g twice daily for 12 weeks) plus L-T4 at the appropriate recommended dosage based on thyroid function significantly decreased TSH, TPOAb, and TGAb levels compared to L-T4 alone [68]. An RCT with 30 HT patients reported that *Bupleurum inula* Flower Soup (one decoction per day divided into two equal doses, twice daily) plus L-T4 decreased serum TPOAb and TGAb compared to L-T4 alone after an 8-week treatment. After a 24-week follow-up, the dose of L-T4 in the group of patients treated with the decoction was lower than before treatment [52]. Similarly, in another RCT with 60 HT patients, Jianggui-Yiying decoction (100 mL twice daily) plus L-T4 could significantly decrease the serum levels of TSH, TPOAb, and TGAb, and increase the levels of FT3 (Free triiodothyronine) and FT4 (Free thyroxine) compared to L-T4 alone after a 12-week treatment [69]. Similarly, other Chinese medicine prescriptions, including modified Shenling-Baizhu San and Shugan-Jianpi formulas, were reported to provide additional effects to L-T4 in treating HT [70,71]. Based on these and other similar studies, a recent meta-analysis including 1247 patients from 18 RCTs concluded that Chinese Herbal Medicine in combination with Western Medicine may improve outcomes in hypothyroid HT patients compared to Western medicine alone [53].

In an RCT with 132 HT patients and three treatment groups (TCM plus selenium vs. TCM vs. selenium), all three treatments lowered TPOAb and TGAb titers compared to baseline. Notably, Lianyu-Xiaoying decoction (150 mL twice daily for 12 weeks) plus selenium supplementation (100 μg twice daily for 12 weeks) further lowered TPOAb and TGAb titers and serum levels of IL-17 and IFN-γ compared to the other two groups. Lianyu-Xiaoying decoction (150 mL twice daily for 12 weeks) lowered TPOAb and TGAb titers and serum levels of IL-17 and IFN-γ compared to selenium supplementation (100 μg twice daily for 12 weeks) alone. These observations suggest that the treatment may inhibit the progression of HT by regulating the immune system [72]. Similar studies with the Qinggan-Sanjie-Xiaoying formula and Qiyu-Yiqi-Shugan recipe were also reported that the efficacy of the TCM plus selenium group was also reported to be superior to that of selenium alone [73,74].

#### Biological Activities of Herbs Commonly Used in TCM for HT

Among the known combinations of herbs in the above 14 Chinese medicine prescriptions, the most frequently used ones were identified (i.e., those present in ≥4 TCM prescriptions in Table 3). Then, the literature was searched for information on the antioxidant, anti-inflammatory, immunomodulatory, and antiaging activities of these herbs; information was available about other organs, but no information specific to the thyroid was found [75,76,77,78,79,80,81,82,83,84,85,86,87,88,89,90] (Table 4). The three most frequently recurring herbs were *Prunella*, *Astragalus membranaceus*, and *Cyperus rotundus*. *Prunella* is a genus of perennial herbaceous plants in the Labiatae family, following a several thousand-year history as a traditional antipyretic and antidotal Chinese herb. Modern pharmacological studies have revealed that *Prunella* possesses antioxidant, immunoregulatory, anti-inflammatory, and antiaging functions [75,76]. The active components related to these functions are mainly triterpenoids, phenolic acids, flavonoids, and polysaccharides. *Astragalus membranaceus* dried root extract, also known as *Astragali radix*, is used in TCM as a tonic remedy. *Astragalus membranaceus* extract attenuates inflammation and oxidative stress in intestinal epithelial cells; it also has other bioactive functions, such as immunomodulatory and antiaging effects [77,78]. *Cyperus rotundus* has a long history of clinical medication and is known as the “holy medicine” of gynecology. Modern pharmacological studies have shown that *Cyperus rotundus* has a wide range of pharmacological activities, including antioxidant and anti-inflammatory effects [79].

### 3.3. Human Studies on Medicinal Herbs for HT

Herbs with pharmaceutical properties are very attractive as natural sources of ingredients to develop novel preventive and therapeutic strategies. Several medicinal herbs are commonly applied in TCM for HT, such as *Cordyceps* (described previously as Jinshuibao Capsule or Bailing Capsule), *Prunella*, *Astragalus membranaceus*, and *Dioscorea nipponica makino*; the results of RCTs and meta-analyses have indicated protective effects of these medicinal herbs in HT [60,91,92,93] (Table 5).

*Cordyceps* refers to the dried fungus *Cordyceps sinensis* that grows on caterpillar larvae. The Bailing Capsule or Jinshuibao Capsule is a proprietary Chinese medicine that contains *Cordyceps sinensis*; it has been applied clinically in polycystic ovary syndrome, diabetic kidney disease, and HT [60,94,95]. Studies have supported the beneficial effects of *Cordyceps* extracts in HT, with satisfactory safety; this efficacy may be attributed to the major bioactive constituents of the extracts, including *Cordycepin* [96]. *Cordycepin* is the major functional component of *Cordyceps sinensis* and has been shown to possess many pharmacological properties, such as antioxidant, anti-inflammatory, antiaging, immunomodulatory, and anti-tumor effects [96,97,98]. A meta-analysis including 14 RCTs with a total of 1014 patients with HT reported that *Cordyceps* preparations in combination with other treatments (iodine-restricted diet or L-T4) may decrease thyroid autoantibodies and inflammatory responses. In HT patients with hypothyroidism, the combination of *Cordyceps* preparations with L-T4 also resulted in higher FT4 levels [60]. These results suggested that *Cordyceps* may be useful in the treatment of HT.

*Prunella*, also named Xiakucao in Chinese, is the dried fruit spikes of *Prunella vulgaris* L., and it has a long history as a herbal medicine for thyroid diseases in TCM. *Prunella* Oral Liquid, or *Prunella* Granule prepared from its extract, has recently been adopted as an adjunctive treatment for patients with HT. Studies support that its use improves thyroid function, decreases thyroid autoantibody titers, and ameliorates clinical symptoms; these effects may be mediated at least partially via counteracting oxidative stress and inhibiting immune reactions and excessive apoptosis [99]. In a meta-analysis consisting of 11 RCTs with a total of 1215 HT patients, *Prunella* preparations combined with L-T4 were found to improve clinical efficiency in terms of decreasing serum TPOAb titers and thyroid volume [91]. Serious adverse events caused by *Prunella* preparations were rarely reported, though mild to moderate gastrointestinal discomfort occasionally occurred, but without statistical significance between treatment and control groups [91,99].

*Astragalus membranaceus*, also named Huangqi in Chinese, corresponds to the dried roots of *Astragalus membranaceus* (Fisch.) Bge. and it has been widely used in treating HT in TCM; beneficial effects have been reported against inflammation, thyrocyte damage, and apoptosis [92,100]. An RCT with 60 HT patients undergoing a 12-week treatment with Huangqi Capsule (0.6 g three times daily) plus iodine-restricted diet compared to iodine-restricted diet alone reported that the combination treatment resulted in a significant reduction in TPOAb and TGAb titers compared to the iodine-restricted diet-only group [92]. These *Astragalus membranaceus*-containing components rich in selenium [101] could increase the effectiveness of the gastrointestinal barrier [102] and, thus, ultimately increase the bioavailability of selenium. The possible effect of *Astragalus membranaceus* on intestinal microbiota can be another potential mechanism that may ameliorate the immune response [103]. Specifically, *Astragalus membranaceus* polysaccharides can increase the abundance of beneficial bacteria, such as Bifidobacterium and Lactobacillus, while reducing harmful bacteria like Shigella [104,105,106]. These changes in bacterial populations can increase the production of short-chain fatty acids like butyrate that strengthen the intestinal barrier and reduce inflammation [107]. Further research is warranted so as to delineate the exact mechanisms underlying these anti-inflammatory effects of microbiota changes.

Similarly, the dried rhizome of *Dioscorea nipponica makino* (“Chuanshanlong” in Chinese) is a medicinal herb that is produced in many areas. Chinese herbal medicine *Dioscorea nipponica makino* granule prepared from its extract has recently been adopted as an adjunct therapeutic for patients with thyroid diseases like HT; the results show that it reduced thyroid autoantibody titers and modulated the immune imbalance [93]. As reported in an RCT with 64 HT patients, a 6-month treatment with Chuanshanlong Granule (10 g, twice daily) plus L-T4 resulted in a significant reduction in TPOAb and TGAb titers, Th17 cells and relevant cytokines compared to L-T4 alone, as well as in a significant increase in the Treg/Th17 ratio [93].

### 3.4. Preclinical Studies on Phytochemicals for HT

TCM formulas and medicinal herbs contain numerous bioactive substances. This contrasts with Western medicine, which generally relies on single compounds. TCM prescriptions consist of various medicinal minerals or herbs, with one considered the principal component and others serving as adjuvants to augment the effects of the principal component or facilitate its delivery. However, individual bioactive compounds in most TCM prescriptions have not yet been identified. Therefore, it is also of interest to discuss mixtures of bioactive phytochemicals that have been extracted from components of TCM. Research studies using animal models of HT have specifically addressed the potential therapeutic effects of Saikosaponin-d (SSd), total Glucosides of *Paeonia lactiflora*, Ginsenosides, and *Tripterygium wilfordii* multiglycosides, which are discussed below [108,109,110,111] (Table 6).

Saikosaponin-d (SSd), extracted from the root of *Bupleurum*, is a highly active triterpenoid saponin that is arguably the most studied in Chinese medicine [112]. SSd has anti-inflammatory, immunoregulatory, anti-tumoral, and other functions. Thanks to its anti-inflammatory and immunoregulatory activities, SSd has been widely used and studied in various autoimmune diseases [113]. An animal study showed that treatment with SSd attenuated the lymphocytic infiltration in the thyroid tissues of a mouse model of HT and reduced serum TPOAb titers; potential mechanisms discussed in the study included regulation of Th1/Th2 and Th17/Treg imbalances, promoting the M2 polarization of macrophages and the associated IL-4- and IL-13-induced anti-inflammatory and tissue repair effects [108].

Total Glucosides of *Paeonia lactiflora* (TGPL) comprise the main bioactive components extracted from *Paeonia lactiflora*, which has been associated with anti-inflammatory, immunomodulatory, and hepatoprotective activities [114,115]. TGPL has thus been proposed for preventing and treating autoimmune diseases like systemic lupus erythematosus, psoriasis, and HT [116,117,118]. Preclinical investigations in a rat model of autoimmune thyroiditis have reported that TGPL significantly reduced serum levels of TPOAb, TGAb, and TNF-α, increased serum levels of IL-10, and alleviated thyroid follicle damage. It is possible that their effect on HT might be related, at least in part, to an improved composition and diversity of the intestinal flora and decreased intestinal mucosal barrier damage [109]. Even though there are no studies that directly link the intestinal microbiota and thyroid autoimmune status and functions, there are some studies that show a potential association [119]. Briefly, some microbiota, such as *Lactobacillus*, *Bifidobacterium*, and *Helicobacter pylori*, can induce thyroid autoimmunity as some bacterial proteins have structural homology with human TPO and TG [120]. However, no solid data show a direct link between microbiota species and thyroid autoimmunity. Last, data indicate that microbiota may affect thyroid function by also regulating the uptake of iodine and selenium [121]. More targeted studies are needed to determine if specific changes in microbiota species have an effect on thyroid function and autoimmune response.

Ginsenosides are the main pharmacologically active ingredients of *Ginseng*. Because studies have shown that they can exert a protective effect against inflammation, oxidative stress, and apoptosis, Ginsenosides have been proposed for the treatment of various diseases [122]. In rat models of autoimmune thyroiditis, Ginsenosides have been reported to reduce the serum titers of thyroid autoantibodies; underlying mechanisms may involve reducing the expression of IL-2, increasing the expression of IL-4, and improving the Th1/Th2 balance [110,123].

*Tripterygium wilfordii* multiglycosides (TWM), extracted and purified from the peeled roots of *Tripterygium wilfordii* Hook F., is a herbal medicine used in TCM for rheumatoid arthritis in relation to its immunosuppressive and anti-inflammatory effects [124]. Preclinical research suggests that TWM may also be a promising thyroid-protective therapy in HT [111]. In a rat model of autoimmune thyroiditis, Tripterygium glycosides were found to reduce thyroid autoantibody titers and improve thyroid function. These effects may be related to regulating the secretion of inflammatory factors such as IL-10 and improving TAC [111].

By focusing on extract mixtures of specific pharmacological classes, preclinical studies such as the above help to bridge the gap between TCM and Western medicine in HT. Further evidence on the anti-inflammatory, immunoregulatory, and antioxidant effects of TCM components could be useful in devising rational approaches to modifying the disease course of HT.

**Table 6:** Preclinical studies on main phytochemicals (mixtures) for HT.

No.	Phytochemicals (Extracts)	Sources of TCM (Plants)	HT Animal Model	Experimental Groups	Interventions	Treatment Duration	Effect	Potential Mechanism	Ref.
1	Saikosaponin-d (SSd)	*Bupleurum*	mouse	16 (HT) vs. 10 (HT) vs. 10 (healthy)	SSd dissolved in sodium carboxymethyl cellulose (CMC) solution vs. sodium CMC solution vs. normal saline, respectively	6 weeks	TPOAb↓	IFN-γ↓, IL-17↓	[108]
2	Total Glucosides of *Paeonia lactiflora* (TGPL)	*Paeonia lactiflora*	rat	24 (HT) vs.8 (HT) vs. 8 (HT) vs. 8 (healthy)	TGPL (low, medium, high dose) vs. selenium vs. normal saline vs. normal saline, respectively	6 weeks	TPOAb↓, TGAb↓	TNF-α↓, IL-10↑	[109]
3	Ginsenosides	*Ginseng*	rat	24 (HT) vs. 8 (HT) vs. 8 (healthy)	Ginsenosides (low, medium, high dose) vs. normal saline vs. normal saline, respectively	8 weeks	TPOAb↓, TGAb↓	IFN-γ↓, IL-2↓, IL-4↑	[110,123]
4	*Tripterygium wilfordii* Multiglycosides (TWM)	*Tripterygium wilfordii* Hook F.	rat	17 (HT) vs. 17 (HT) vs. 17 (HT) vs. 17 (healthy)	TWM vs. selenium vs. normal saline vs. normal saline, respectively	4 weeks	TPOAb↓, TGAb↓, FT3↑, FT4↑	TNF-α↓, IL-10↑, SOD↑, GPx↑, TAC↑	[111]

## 4. Conclusions

Persistent symptoms, life-long dependence on levothyroxine substitution therapy, and other factors may result in low HRQoL in patients with HT. Targeted interventions to modify the clinical course of HT, especially during its early stages, might delay or prevent progression to hypothyroidism, thereby helping to preserve the patient’s HRQoL. Therefore, a better understanding of the pathophysiological mechanisms involved in HT initiation and progression (including oxidative stress, inflammation, immune imbalance, etc.) may facilitate rational strategies to prevent the irreversible destruction of the thyroid parenchyma. The present review suggests that a substantial body of evidence from RCTs, systematic reviews, and meta-analyses indicates that TCM formulas and medicinal herbs can exert promising protective effects against HT. Evidence from the study of these approaches in other organs suggests that anti-inflammatory, immunoregulatory, and antioxidant effects may be involved. Further, we found that evidence from preclinical studies also supports the potential disease-modifying effects of TCM bioactive compound mixtures or phytochemicals used in TCM. Based on these observations, we suggest that further structured preclinical and clinical research into TCM could be beneficial to cross-validate therapeutic approaches to the treatment of HT. Given the broad anti-inflammatory, immunoregulatory, and antioxidant effects of many TCM components, parallel investigations into their underlying mechanisms in HT preclinical models and clinical studies are highly relevant. Finally, given the above, we are strongly in favor of closer collaboration between researchers and practitioners of TCM and Western medicine for the benefit of patients with HT specifically and with other thyroidal and non-thyroidal diseases more generally.

## Figures and Tables

**Table 1:** A typical TCM syndrome score form for HT.

Common TCM Symptoms	Absent (0)	Mild (2)	Moderate (4)	Severe (6)
1. Anterior neck enlargement	Absent	Enlargement is palpable but not visible	Enlargement is both visible and palpable, but it does not exceed the sternocleidomastoid muscle	Enlargement beyond the sternocleidomastoid muscle
2. Cold intolerance	Absent	Aversion to wind	Aversion to cold but without need to add extra clothes	Aversion to cold, with need to add extra clothes
3. Epigastric pain	Absent	Occasional	Pain for less than 2 h per day	Persistent pain
4. Emotional instability	Absent	Occasional	Frequent but manageable	Frequent and difficult to control
5. Fatigue	Absent	Fatigue after strenuous activity	Fatigue after light activity	Fatigue at the resting stage
6. Loss of appetite	Absent	Occasional	Eating 1/3 to one-half less than usual	Eating less than one-half of usual
7. Sleepiness	Absent	Mildy sleepy	Moderately sleepy	Overtly sleepy
8. Insomnia	Absent	Occasional	Frequent	Almost daily
9. Constipation	Absent	Difficulty in defecation, one bowel movement per day	Difficulty in defecation, one bowel movement every 2–3 days	Difficulty in defecation, one bowel movement every more than 3 days
10. Edema	Absent	Facial or lower extremity edema	Facial and lower extremity edema	Generalized edema

**Table 2:** Typical categories of clinical efficacy of TCM for HT.

Category	Definition
1. Clinical cure	Disappearance or near disappearance of clinical symptoms and signs in TCM and reduction in TCM syndrome score by ≥95%
2. Obvious effect	Significant improvement of clinical symptoms and signs, reduction in TCM syndrome score by 70–95%
3. Effective	Improvement of clinical symptoms and signs in TCM, reduction in TCM syndrome score by 30–70%
4. Ineffective	No obvious improvement in clinical symptoms and signs, or even aggravation, and reduction in TCM syndrome score < 30%.

**Table 3:** Main Chinese medicine prescriptions for HT in human studies.

No.	TCM Formula	Constituents	Study Design	Thyroid Function	Interventions (Sample Size)	Treatment Duration	TCM Effects	Potential Mechanisms	Ref.
1	Fuzheng-Jiedu-Xiaoying decoction	*Astragalus membranaceus*, *pangolin*, *Lonicera japonica*, *Prunella*, *Angelica sinensis*, *Forsythia suspensa*, *Scutellaria barbata*, *Buthus martensii karsch*, *Trionyx sinensis wiegmann*, *Glycyrrhiza uralensis*	Randomized, controlled	/	TCM (n = 30) vs. Selenium (n = 30)	12 weeks	TPOAb↓, TGAb↓, FT3↑, FT4↑, TSH↓, TCM syndrome score↓, total effective rate↑, thyroid volume↓	Treg ratio↑, Th17 ratio↓, Th17/Treg ratio↓, IL-17↓, IL-6↓	[63]
2	Jianpi-Xiaoying decoction	*Astragalus membranaceus*, *Atractylodes macrocephala*, *Codonopsis pilosula*, *Poria cocos*, *Curcuma phaeocaulis*, *Sinapis alba*, *Arctium lappa*, *Prunella*, *Hedyotis diffusa*, *Euphorbia humifusa*, *Cyperus rotundus*, *Epimedium brevicornu*, *Ganoderma lucidum*, *Glycyrrhiza uralensis*	Randomized, controlled	/	TCM (n = 40) vs. Selenium (n = 40)	12 weeks	TPOAb↓, TGAb↓, TCM syndrome score↓, total effective rate↑	IFN-γ↓, IL-4/IFN-γ ratio↑	[64]
3	Qijian-Goiter-eliminating decoction	*Astragalus membranaceus*, *Euonymus alatus*, *Dioscorea nipponica makino*, *Paeonia ostii*	Randomized, controlled	Euthyroid	TCM (n = 30) vs. Selenium (n = 30)	12 weeks	TPOAb↓, TGAb↓, thyroid volume↓, TCM syndrome score↓, total effective rate↑	MDA↓, SOD↑, GPx↑, TAC↑, IL-1β↓, IL-18↓	[65]
4	Qiaojiafang granule	*Astragalus membranaceus*, *Prunella*, *Atractylodes macrocephala*, *Forsythia suspensa*, *Rehmannia*, *Cyperus rotundus*	Randomized, controlled	Euthyroid	TCM (n = 35) vs. Selenium (n = 35)	12 weeks	TPOAb↓, TGAb↓	/	[66]
5	Shugan Sanjie formula	*Bupleurum*, *Cyperus rotundus*, *Prunella*, *Fritillaria*, *Paeonia ostii*, *Glycyrrhiza uralensis*	Randomized, controlled	Euthyroid	TCM (n = 60) vs. Placebo (n = 60)	12 weeks	TPOAb↓, TGAb↓, FT3↑, FT4↑, TSH↓, TCM syndrome score↓, total effective rate↑, thyroid volume↓	/	[67]
6	Jieyu-Xiaoying decoction	*Dioscorea nipponica makino*, *Ostrea gigas*, *Hedyotis diffusa*, *Bupleurum*, *Angelica sinensis*, *Paeonia ostii*, *Dioscorea opposita thunb*, *Cyperus rotundus*, *Atractylodes macrocephala*, *Poria cocos*, *Scutellaria barbata*, *Pseudostellaria heterophylla*, *Sparganium stoloniferum*, *Curcuma phaeocaulis*, *Scutellaria baicalensis*, *Pinellia ternata*, *Fritillaria thunbergii*	Randomized, controlled	/	TCM + Selenium (n = 47) vs. Selenium (n = 47)	12 weeks	TPOAb↓, TGAb↓, TSH↓, thyroid volume↓, TCM syndrome score↓, total effective rate↑	IFN-γ↓, IL-17↓, IL-10↑, IL-35↑	[62]
7	Jinkui-Shenqi pill	*Rehmannia*, *Dioscorea opposita thunb*, *Cornus officinalis*, *Poria cocos*, *Moutan cortex*, *Alisma orientale*, *Cinnamomum cassia presl*, *Aconitum carmichaelii*, *Achyranthes bidentata*, *Plantago asiatica*	Controlled	Hypothyroid	TCM + L-T4 (n = 30) vs. L-T4 (n = 34)	12 weeks	TPOAb↓, TGAb↓, TSH↓, total effective rate↑	/	[68]
8	*Bupleurum inula* Flower Soup	*Bupleurum*, *Paeonia ostii*, *Pinellia*, *Fritillaria*, *Prunella*, *Cyperus rotundus*, *Codonopsis*, *Inula*	Randomized, controlled	Hypothyroid	TCM + L-T4 (n = 24) vs. Placebo + L-T4 (n = 24)	8 weeks	TPOAb↓, TGAb↓, TSH↓, thyroid volume↓, HRQoL score↑	/	[52]
9	Jianggui-Yiying decoction	*Zingiber officinale*, *Cinnamomum cassia*, *Rehmannia*, *Dioscorea opposita thunb*, *Poria cocos*, *Alisma orientale*, *Prunella*, *Scrophularia ningpoensis*, *Astragalus membranaceus*, *Atractylodes macrocephala*, *Angelica sinensis*, *Ligusticum chuanxiong*, *Citrus reticulata blanco*	Randomized, controlled	Hypothyroid	TCM + L-T4 (n = 30) vs. L-T4 (n = 30)	12 weeks	TPOAb↓, TGAb↓, FT3↑, FT4↑, TSH↓, TCM syndrome score↓	/	[69]
10	Modified-Shenling-Baizhu San	*Panax ginseng*, *Atractylodes macrocephala*, *Poria cocos*, *Glycyrrhiza uralensis*, *Dolichos lablab*, *Dioscorea opposita thunb*, *Coix lacryma-jobi*, *Nelumbo nucifera*, *Amomum villosum*, *Platycodon grandiflorum*, *Scrophularia ningpoensis*, *Fritilaria thunbergii*, *Ostrea gigas*, *Prunella*, *Cremastra appendiculata*	Randomized, controlled	Hypothyroid	TCM + L-T4 (n = 34) vs. L-T4 (n = 34)	12 weeks	TPOAb↓, TGAb↓, FT3↑, FT4↑, TSH↓, TCM syndrome score↓, total effective rate↑	/	[70]
11	Shugan-Jianpi formula	*Bupleurum*, *Atractylodes macrocephala*, *Astragalus membranaceus*, *Paeonia ostii*, *Prunella*, *Sparganium stoloniferum*, *Cyperus rotundus*, *Citrus reticulata blanco*, *Glycyrrhiza uralensis*	Randomized, controlled	Hypothyroid	TCM + L-T4 (n = 41) vs. L-T4 (n = 41)	4 weeks	TPOAb↓, TGAb↓, FT3↑, FT4↑, TSH↓, TCM syndrome score↓, total effective rate↑	IFN-γ↓, TNF-α↓, IL-8↓, IL-6↓	[71]
12	Lianyu-Xiaoying decoction	*Bupleurum*, *Cyperus rotundus*, *Curcuma wenyujin*, *Paeonia ostii*, *Atractylodes macrocephala*, *Poria cocos*, *Pinellia ternata*, *Fritilaria thunbergii*, *Dioscorea nipponica makino*, *Hedyotis diffusa*, *Paris polyphylla*, *Scutellaria barbata*, *Glycyrrhiza uralensis*	Randomized, controlled	Euthyroid	TCM + Selenium (n = 44) vs. TCM (n = 44) vs. Selenium (n = 44)	12 weeks	TPOAb↓, TGAb↓	IFN-γ↓, IL-17↓	[72]
13	Qinggan-Sanjie-Xiaoying formula	*Prunella*, *Platycodon grandiflorum*, *Paeonia ostii*, *Ostrea gigas*, *Astragalus membranaceus*, *Scutellaria baicalensis*, *Paeonia suffruticosa*, *Hyriopsis cumingii*, *Ranunculus ternatus*, *Bupleurum*, *Albizia julibrissin*	Retrospective	Euthyroid	TCM + Iodine-restricted diet (n = 40) vs. TCM + Selenium + Iodine-restricted diet (n = 40) vs. Iodine-restricted diet (n = 38)	12 weeks	TPOAb↓, TGAb↓, TCM syndrome score↓	/	[73]
14	Qiyu-Yiqi-Shugan recipe	*Astragalus membranaceus*, *Cyperus rotundus*, *Curcuma wenyujin*, *Ganoderma lucidum*, *Hedyotis diffusa*, *Glycyrrhiza uralensis*	Controlled	Euthyroid or Subclinical hypothyroidism	TCM + Selenium (n = 89) vs. TCM (n = 140) vs. Selenium (n = 110)	8 weeks	TPOAb↓, TGAb↓	/	[74]

/ Not specified in the respective articles.

**Table 4:** Main medicinal herbs used in Chinese medicine prescriptions for HT.

No.	TCM Name	Use Frequency (in Table 3)	Medicinal Herbs	Bioactivities	Ref.
1	*Prunella*	9	Dried fruit spikes of *Prunella vulgaris* L.	AO, IM, AI, AA	[75,76]
2	*Astragalus membranaceus*	8	Dried roots of *Astragalus membranaceus* (Fisch.) Bge	AO, IM, AI, AA	[77,78]
3	*Cyperus rotundus*	8	Dried rhizomes of *Cyperus rotundus* L.	AO, AI	[79]
4	*Paeonia ostii*	7	Dried roots of *Paeonia lactiflora* Pall.	AO, IM, AI	[80]
5	*Atractylodes macrocephala*	7	Dried rhizomes of *Atractylodes macrocephala* Koidz.	AO, IM, AI	[81,82,83]
6	*Glycyrrhiza uralensis*	7	Dried rhizomes of *Glycyrrhiza uralensis* Fisch.	AO, IM, AI	[84]
7	*Bupleurum*	6	Dried roots of *Bupleurum chinense* DC.	AO, IM, AI, AA	[85,86]
8	*Poria cocos*	6	The dried sclerotium of *Poria cocos* (Schw.) Wolf	AO, IM, AI	[87]
9	*Dioscorea opposita thunb*	4	Dried rhizomes of *Dioscorea opposita* Thunb.	AO, IM, AI, AA	[88,89]
10	*Hedyotis diffusa*	4	Dried whole plants of *Oldenlandia diffusa*(Willd.) Roxb.	AO, IM, AI	[90]

AO: antioxidant; IM: immunomodulatory; AI: anti-inflammatory; AA: antiaging.

**Table 5:** Human studies on main medicinal herbs for HT.

No.	TCM Name	Medicinal Herbs	Study Design	Thyroid Function	Sample Size	Interventions	Treatment Duration	TCM Effects	Potential Mechanisms	Ref.
1	*Cordyceps sinensis*	The dried fungus *Cordyceps sinensis* growing on caterpillar larvae	Meta-analysis	Variable	1014 (14 RCTs)	TCM + Iodine-restricted diet or L-T4 vs. Iodine-restricted diet or L-T4	Variable	TPOAb↓, TGAb↓, FT4↑	TNF-α↓, IL-2↓, IL-6↓	[60]
2	*Prunella*	Dried fruit spikes of *Prunella vulgaris* L.	Meta-analysis	Hypothyroid	1215 (11 RCTs)	TCM + L-T4 vs. L-T4	Variable	TPOAb↓, thyroid volume↓, total effective rate↑	/	[91]
3	*Astragalus membranaceus*	Dried roots of *Astragalus membranaceus* (Fisch.) Bge	Randomized, controlled	/	30 vs. 30	TCM + Iodine-restricted diet vs. Iodine-restricted diet	12 weeks	TPOAb↓, TGAb↓	/	[92]
4	*Dioscorea nipponica makino*	Dried tubers of *Dioscorea nipponica makino*	Randomized, controlled	Hypothyroid	32 vs. 32	TCM + L-T4 vs. L-T4	24 weeks	TPOAb↓, TGAb↓, FT3↑, FT4↑, TSH↓, TCM syndrome score↓, total effective rate↑	Treg ratio↑, Th17 ratio↓, IL-6↓	[93]

/ Not specified in the respective articles.

## Abbreviations

AGEs	advanced glycation end products
AITD	autoimmune thyroid disease
Anti-TG	anti-thyroglobulin
Anti-TPO	anti-thyroid peroxidase
CAT	catalase
CMC	carboxymethyl cellulose
FT3	Free triiodothyronine
FT4	Free thyroxine
GPx	glutathione peroxidase
H_2_O_2_	hydrogen peroxide
HLA-DR	Human Leukocyte Antigen DR
HRQoL	health-related quality of life
HT	Hashimoto’s thyroiditis
IFN-γ	interferon-γ
IL	interleukin
L-T4	levothyroxine
NOX	NADPH oxidases
NRF2	nuclear factor erythroid 2-related factor 2
OSI	oxidative stress index
RCTs	randomized controlled trials
RNS	reactive nitrogen species
ROS	reactive oxygen species
SCI	Science Citation Index
Sec	selenocysteine
SELENOS	selenoprotein S
SNPs	single nucleotide polymorphisms
SOD	superoxide dismutase
T3	triiodothyronine
T4	thyroxine
TAC	total antioxidant capacity
TCM	traditional Chinese medicine
TGAb	anti-thyroglobulin autoantibodies
Th	T helper
TNF-α	tumor necrosis factor-alpha
TOS	total oxidant status
TPOAb	anti-thyroid peroxidase autoantibodies
Treg cells	T regulatory lymphocytes
TSH	thyroid-stimulating hormone
